# Branched-Chain Amino Acids as New Biomarkers of Major Depression - A Novel Neurobiology of Mood Disorder

**DOI:** 10.1371/journal.pone.0160542

**Published:** 2016-08-04

**Authors:** Andreas Baranyi, Omid Amouzadeh-Ghadikolai, Dirk von Lewinski, Hans-Bernd Rothenhäusler, Simon Theokas, Christoph Robier, Harald Mangge, Gerhard Reicht, Peter Hlade, Andreas Meinitzer

**Affiliations:** 1 Department of Psychiatry and Psychotherapeutic Medicine, Medical University of Graz, Graz, Austria; 2 Institute for International Management Practice, ARU Cambridge, Cambridge, UK; 3 Hospital of the Brothers of St. John of God, Graz, Austria; 4 Division of Cardiology, Department of Internal Medicine, Medical University of Graz, Graz, Austria; 5 Clinical Institute of Medical and Chemical Laboratory Diagnostics, Medical University of Graz, Graz, Austria; University of Stellenbosch, SOUTH AFRICA

## Abstract

**Background:**

The proteinogenic branched-chain amino acids (BCAAs) valine, leucine and isoleucine might play an unrecognised crucial role in the development of depression through their activation of the mammalian target of rapamycin (mTor) pathway. The aim of this research project is to evaluate whether BCAAs are altered in patients with major depression and might thus be appropriate biomarkers for major depression.

**Methods:**

The concentrations of valine, leucine and isoleucine were determined in 71 in-patients with major depression and 48 healthy controls by high-pressure liquid chromatography. Psychiatric and laboratory assessments were obtained at the time of in-patient admittance.

**Results:**

The BCAAs are significantly decreased in patients with major depression in comparison with healthy subjects (valine: Mann-Whitney-U: 968.0; p <0.0001, leucine: Mann-Whitney-U: 1246.5; p = 0.013, isoleucine: Mann-Whitney-U: 1252.5; p = 0.014). Furthermore, as shown by Spearman's rank correlation coefficients, there is a significant negative correlation between valine, leucine and isoleucine concentrations and the Hamilton Depression Rating Scale (HAMD-17) as well as Beck Depression Inventory (BDI-II) scores.

**Conclusions:**

Our study results are strong evidence that in patients with major depression, BCAAs might be appropriate biomarkers for depression. Reduced activation of the mammalian target of rapamycin (mTor) due to a reduction of BCAAs might play a crucial unrecognised factor in the etiology of depression and may evoke depressive symptomatology and lower energy metabolism in patients with major depression. In the future, mTor and its up- and downstream signalling partners might be important targets for the development of novel antidepressants.

## Introduction

The proteinogenic branched-chain amino acids (BCAAs) leucine, isoleucine and valine are the most hydrophobic of the amino acids and belong to the nine essential amino acids [1]. According to Muin et al. (2009) BCAA metabolism is directly connected to energy metabolism and oxidative BCAAs degradation leads to Krebs cycle intermediates [2]. Furthermore, BCAAs have anabolic effects on protein metabolism through increasing the rate of protein synthesis and decreasing the rate of protein degradation in resting human muscles. Thus, even during recovery from endurance sports, branched-chain amino acids have anabolic effects in human muscles [3].

Recent study results underline the widely recognised significance of BCAAs as specific biomarkers of health and disease [1]. Thus, previous studies suggest that BCAAs are associated with the risk of cardiovascular disease, end-stage renal failure, and ischemic stroke. In addition, circulating levels of BCAAs may have the potential to predict populations at risk for cardio-metabolic disease and mortality from ischemic heart disease [1]. In patients suffering from cardiovascular diseases BCAAs are associated with mortality after cardiac catheterisation [4]. Circulating concentrations of valine and leucine are decreased in end-stage renal failure [5]. In regard to cerebrovascular diseases, it needs to be mentioned that plasma BCAA levels are significantly decreased in patients with transient ischemic attack or acute ischemic stroke, and low BCAA concentrations deteriorate outcomes in ischemic stroke patients [1]. In addition, BCAA administration has a beneficial effect on hepatic encephalopathy [6]. In a study of Michaliszyn et al. [7] BCAAs were positively associated with beta cell function.

### Mammalian Target of Rapamycin (mTor)

The mammalian target of rapamycin (mTor), also referred to as a mechanistic target of rapamycin, is a ubiquitous serine/threonine protein kinase. mTor regulates cell growth, cell proliferation, cell survival, cell motility, protein synthesis, autophagy and transcription [8]. According to Hay et al. (2004) mTor integrates the input from upstream pathways, including BCAAs (particularly leucine), insulin, and growth factors [8]. A BCAA-induced chronic activation of mTor induces insulin resistance and early beta cell dysfunction [9–11]. Furthermore, mTor is dysregulated during depressive episodes, and ketamine-induced activation of mTor is associated with a short-term decrease of depressive symptoms in patients suffering from major depression [12].

Prior psychiatric research focusing on BCAAs was based only on the competitive impact of large neutral amino acids on the transport system for the serotonin precursor tryptophan across the blood-brain barrier. The clinical relevance of this competitive effect is not fully understood and controversially discussed [13]. However, until now the impact of BCAAs on mTor might have been overlooked in patients suffering from major depression.

### Aims of the Study

BCAAs might play an unrecognised crucial role in the development of depressive symptomatology through their activation of the mTor pathway. Consequently, the aim of this exploratory research project is to evaluate whether BCAAs are altered in patients with major depression and might thus be appropriate biomarkers for major depression.

## Materials and Methods

### Participants and Study Design

This publication is part of our research project on the etiopathogenesis and health consequences of depressive symptomatology in somatically healthy patients with major depression. In total 122 participants were recruited for this research project. In our former study [14] we described the nitric oxide-related biological pathways in patients with major depression. As this former study [14] has described potential somatically relevant negative health consequences of depression, this current study in contrast aims to investigate the etiopathogenesis of depression using an innovative approach, based on the activation of the mTor pathway. The details of the project and the recruitment procedure are described elsewhere [14] but are repeated in detail in this research article to facilitate understanding and interpretation of the new study results. As previously described, out of the 119 study participants a total of 71 suffered from major depression, 48 participants were physically and mentally healthy and without a former history of psychiatric disorders, and 3 individuals refused to participate in this research project after signing the informed consent form. All patients with major depression were in-patients, treated in the Department of Psychiatry, Hospital of the Brothers of St. John of God, Graz, Austria. As previously described, all healthy controls were recruited using flyers distributed throughout the city of Graz. The flyers briefly introduced the study and provided contact details of the study’s staff.

In our research project the following reasons for exclusion from enrolment applied to the patients with major depression and the healthy controls: a.) pregnancy, b.) significant co-morbid conditions (e.g. cardio-vascular illness, chronic kidney disease, cancer), c.) disease or drugs that influence the immune system, d.) signs of infection, e.) diagnosis of a neurological disease, and f.) fasting diet or dietary supplements [14].

Regarding the subgroup of depressive patients, all psychiatric and biological assessments were carried out at the time of in-patient admittance to the Department of Psychiatry, Hospital of the Brothers of St. John of God, Graz, Austria, due to major depression.

Our research project and the current study have been approved by the Institutional Review Board of the Medical University of Graz. Data protection met the standards set by Austrian law. All study participants had to give signed informed consent, and could decide to withdraw from this research project at any time. The presented study has been carried out according to the GCP standards and the Declaration of Helsinki.

### Biological Assessments

For all fasting study participants (subjects with major depression at the time of in-patient admittance and the somatically healthy controls) blood was sampled between 08.00 and 09.00.

The BCAAs leucine, isoleucine and valine were measured with modifications of previously described chromatographic methods [15, 16]. Briefly, after precipitation of EDTA plasma with perchloric acid following neutralisation of the supernatant with sodium carbonate, the extracted amino acids were derivatised with o-phtalaldehyde and separated on a reversed phase column with gradient elution. Quantification was performed with ratios of fluorescence signals of leucine, isoleucine and valine to the internal standard norvaline in comparison to the appropriated calibration curves. Intra-assay and inter-assay CVs were all below 10%.

### Psychiatric Assessments

As previously described [14] all participants were evaluated in a blinded clinical interview performed by experienced consultation-liaison psychiatrists (O.A.-G, A.B.), and supported by the results of the observer-rating Hamilton Depression Scale (HAMD-17 [17]) and the self-rating Beck Depression Inventory scale (BDI-II [18]). The results were used to identify depressive symptomatology (Inter-rater reliability for HAMD-17: Spearman’s rank correlation coefficient, r = 0.994).

1. **Author-Compiled Sociodemographic Questionnaire.** Demographic data collected for this research project were age, gender, marital status, employment status, and living arrangements at the time of psychiatric assessment [14].
2. **Hamilton Depression Scale (HAMD-17 [17]).** The observer-rating scale HAMD-17 consists of 17 items and is an established measure of the severity of depressive symptomatology by examining mood, suicide ideation, feelings of guilt, psychomotor agitation or retardation, anxiety, insomnia and somatic symptoms [17].
3. **Beck Depression Inventory (BDI-II [18]).** The self-rating questionnaire BDI-II consists of 21 questions regarding significant depressive symptoms such as sadness, hopelessness and irritability, cognitions such as guilt or feelings of being punished, and physical symptoms of depression. The total values of the BDI-II range between 0 and a maximum of 63 points [18].

### Statistical Analyses

All descriptive statistics regarding sociodemographic, biochemical and psychometric data are presented as mean and standard deviation (SD). χ^2^ tests were used to evaluate group differences in sociodemographic categorical variables. The BCAAs were not normally distributed and we therefore applied the non-parametric Mann-Whitney-U test. To counteract the problem of multiple comparisons we also performed the Bonferroni correction in comparing the levels of the amino acids between depressed patients and normal control subjects. Spearman’s rank correlation coefficients between valine, leucine and isoleucine concentrations and the HAMD-17 as well as BDI-II scores have been calculated.

All statistical tests were two-tailed, with significance set at p<0.05. The statistical analyses were performed with SPSS 23.0 for Windows (SPSS; Chicago, IL).

## Results

### Sociodemographic, Clinical and Treatment Characteristics

The basic sociodemographic, clinical (BDI-II-, HAMD-17 scores) and treatment characteristics of the participating 71 in-patients with major depression and the 48 healthy controls of our research project have already been described in detail in our previous study concerning nitric oxide-related biological pathways [14]. However, for an easier interpretations of the new data, the sociodemographic and clinical characteristics are summarised in **Table 1**. No patient with major depression had additional psychotic symptoms. All participants were of caucasian origin.

**Table 1:** Sociodemographic and clinical characteristics of the participants [14].

Category		Major Depression (n = 71)	Healthy Controls (n = 48)	p
**Gender**	Male	48 (67.6%)	31 (64.6%)	χ^2^ = 0.117; df = 1; p = 0.732^a^
	Female	23 (32.4%)	17 (35.4%)	
**Age**	Mean	49.15	46.06	Mann-Whitney-U-Test: 1423.0; p = 0.13
	SD	11.35	18.31	
	Median	50.00	40.00	
	Range	56.00	55.00	
**Marital status**	Single	16 (23.2%)	17 (35.4%)	χ^2^ = 2.608; df = 3; p = 0.456^a^
	Partner	48 (69.6%)	28 (58.3%)	
	Widowed	2 (2.9%)	2 (4.2%)	
	Divorced	3 (4.3%)	1 (2.1%)	
**Employment status**	Paid work	32 (46.4%)	23 (47.9%)	χ^2^ = 0.27; df = 1; p = 0.870^a^
	No paid work (homeworker, unemployed, retired)	37 (53.6%)	25 (52.1%)	
**Living arrangements**	Alone	18 (26.1%)	11 (22.9%)	χ^2^ = 0.153; df = 1; p = 0.696^a^
	With others (family, partner or friends)	51 (73.9%)	37 (77.1%)	
**Length of acute depressive episode** (months)	Mean	5.51		
	SD	2.66		
	Median	5.00		
	Range	16		
**Number of depressive episodes**	Mean	2.61		
	SD	1.65		
	Median	2		
	Range	7		
**Overall duration since first depressive episode** (months)	Mean	63.27		
	SD	76.23		
	Median	36		
	Range	359		

Legend

SD = Standard deviation

^a^ χ^2^ test

Patients with major depression at the time of in-patient admittance to the Department of Psychiatry were treatment-resistant to current standard dosis antidepressants therapy. The length of psychopharmacological treatment before in-patient admittance has been at a mean of 12.79 months (S.D.: 25.67; Median: 6.00 months; Range: 192). The antidepressants at the time of in-patient admittance are listed in **Table 2.**

**Table 2:** Antidepressants before in-patient admittance.

Antidepressants				
	Number of patients	Median (mg)	Minimum (mg)	Maximum (mg)
Sertraline	12 (16.9%)	50	25	200
Escitalopram	11 (15.5%)	15	10	20
Paroxetine	3 (4.2%)	40	20	40
Fluoxetine	3 (4.2%)	40	40	40
Mirtazapine	6 (8.5%)	30	15	45
Venlafaxine	11 (15.5%)	225	150	300
Duloxetine	12 (16.9%)	75	30	90
Trazodone	24 (33.8%)	150	25	300
Amitryptiline	1 (1.4%)	25	25	25
Bupropion	5 (7%)	150	150	300

**Table 3** shows the HAMD-17 and BDI-II scores for the participants with major depression at the time of in-patient admittance to the Department of Psychiatry and the somatically healthy controls.

**Table 3:** HAMD-17 and BDI-II scores for the patients with major depression and healthy controls [14].

		Patients with Major Depression (n = 71)	Healthy Controls (n = 48)	p
**HAMD-17**	Mean	21.07	0.42	Mann-Whitney-U: 0.0; **p<0.0001**^a^
	SD	4.89	1.01	
	Median	20.00	.00	
	Range	21.00	5.00	
**BDI-II**	Mean	24.47	1.52	Mann-Whitney-U: 9.5; **p<0.0001**^a^
	SD	9.69	1.92	
	Median	24.00	1.00	
	Range	39.00	8.00	

Legend

^a^ Mann-Whitney-U-Test

BDI-II: Beck Depression Inventory

HAMD-17: Hamilton rating scale for depression

### Branched-Chain Amino Acids

The BCAAs valine, leucine and isoleucine are significantly decreased in patients with major depression in comparison with healthy subjects. **Table 4** shows the valine, leucine and isoleucine concentrations for the patients suffering from major depression and the healthy controls.

**Table 4:** Biological assessments for the patients with major depression at the time of in-patient admittance to the Department of Psychiatry and healthy controls.

		Patients with Major Depression (n = 71)	Healthy Controls (n = 48)	p
**Valine, μ**mol/L	Mean	208.69	241.74	Mann-Whitney-U: 968.0; **p<0.0001**^**a**^
	SD	36.09	45.35	
	Median	208.36	238.00	
	Range	145.40	216.41	
**Leucine, μ**mol/L	Mean	121.21	132.35	Mann-Whitney-U: 1246.5; **p = 0.013**^**a**^
	SD	24.94	21.87	
	Median	118.83	130.24	
	Range	104.31	100.45	
**Isoleucine, μ**mol/L	Mean	62.11	67.11	Mann-Whitney-U: 1252.5; **p = 0.014**^**a**^
	SD	13.31	12.14	
	Median	60.62	65.83	
	Range	58.46	58.19	

Legend

^a^ Mann-Whitney-U-Test, Bonferroni correction α = 0.017

BDI-II: Beck Depression Inventory-II

HAMD-17: Hamilton rating scale for depression

Furthermore, as shown by Spearman’s rank correlation coefficients there was a significant negative correlation between valine, leucine and isoleucine concentration and the HAMD-17 as well as BDI-II scores. **Table 5** provides the Spearman’s rank correlation coefficients between branched-chain amino acids and HAMD-17 and BDI-II scores.

**Table 5:** Spearman's rank correlation coefficients between branched-chain amino acids and Hamilton Depression Rating Scale (HAMD-17) and Beck Depression Inventory (BDI-II) scores.

	HAMD-17		BDI-II	
	r	p	r	p
**Valine**	-0.34	**p <0.0001**	-0.33	**p <0.0001**
**Leucine**	-0.23	**p = 0.012**	-0.22	**p = 0.018**
**Isoleucine**	-0.22	**p = 0.018**	-0.22	**p = 0.017**

Legend

BDI-II: Beck Depression Inventory-II

HAMD-17: Hamilton rating scale for depression

## Discussion

In the current study the three BCAAs valine, leucine and isoleucine were significantly decreased in patients with major depression. In addition, there was a significant negative correlation between valine, leucine and isoleucine concentration and the HAMD-17 as well as BDI-II scores. These findings are consistent with the results of our former study about IFN-α induced depressive symptomatology [19]. In this previous study the sum of the competing amino acids valine, isoleucine, leucine, tyrosine and phenylalanine also significantly declined in patients with IFN-α induced depression. Furthermore, Capuron et al. [20] reported that one month after the onset of IFN-α treatment the sum of the large neutral amino acids was significantly lower when compared to baseline. In an animal study by Webhofer et al. (2011) [21], the concentrations of the essential amino acids valine, leucine and isoleucine increased by 50–70% in mice after chronic treatment with the antidepressant paroxetine. Aquilani et al. reported in their former study that alterations in Krebs cycle intermediates due to oxidative BCAA degradation may impact on neurotransmitter synthesis. These findings suggest that increased BCAA levels may influence the synaptic transmission [12]. In addition, the large neural amino acids and the serotonin precursor tryptophan make use of the same transport system across the blood-brain barrier. As a consequence, a competition among these amino acids for the carrier protein might be the result [22]. Thus, increased concentrations of large neural amino acids might be associated with reduced tryptophan availability in the brain for serotonin synthesis. However, the clinical impact of the competitive effect of BCAAs is controversially discussed [13], and the decrease of BCAAs in depressive patients observed in our study could lead to the alternative hypothesis that the mTor pathway might be dysregulated due to BCAA deficiency during depressive episodes. Thus, a sub-anaesthetic dosis of ketamine activates mTor rapidly and is associated with a fast decrease of depressive symptomatology in patients with major depression [23]. Furthermore, some case studies report that the mTor pathway is activated in the peripheral blood of depressed patients after the acute administration of ketamine [24]. In addition, Diaz Granados et al. [25] reported that a single infusion of ketamine decreased suicidal ideation scores in treatment-resistant patients with major depression disorder within 40 minutes. Ketamine is well known as an antagonist of the NMDA receptor. It interacts with channels and with voltage-sensitive Ca2+ opioid, monoamine and muscarinic receptors [26]. As a consequence of an administration of ketamine, synaptic signalling proteins and the number and function of new spine synapses in the prefrontal cortex increased in an animal study with rats [27, 28]. In addition, Chandran et al. reported that chronic stress exposure decreases the phosphorylation levels of mTor and its downstream signalling components in the amygdala and causes brain region-specific abnormalities in signalling pathways [29]. The mTor pathway might also be influenced by other antidepressants besides ketamine. For example, in an animal study with rats a combination of fluoxetine and methylphenidate induced mTor activity [30]. Furthermore, in rat embryonic fibroblasts, the SSRI sertraline exerted anti-proliferative activity by targeting the mTor signalling pathway [26]. In contrast, inhibition of mTor by rapamycin reverses the antidepressant effects of ketamine in patients with major depression [31]. These findings suggest that an activation of mTor is associated with a decrease of depressive symptomatology in patients suffering from depression. The BCAAs, especially leucine, are significant activators of mTor. As the results of this study highlight significant lower plasma concentrations of BCAAs in patients with major depression in comparison with healthy subjects, we hypothesise that mTor in depressive patients is less activated due to BCAA deficiency, resulting in depressive symptomatology and lower energy metabolism due to Krebs cycle intermediates.

### Limitations

In general, in healthy non-psychiatric subjects fasting blood BCAAs levels seem to be stableover time in a narrow range. In our present exploratory study BCAAs are significantly lower in patients with major depression in comparison with healthy controls. The observed BCAAs levels in depressed patients might reflect a specific phenotype based on the interplay of imbalanced enzymatic systems. An alternative hypothesis might be that our study results represent a compensatory homeostatic response, particularly given the treatment status. However, contradicting this hypothesis the valine, leucine and isoleucine levels strongly increased in mice after chronic treatment with paroxetine [21]. These study results concerning the impact of paroxetine on BCAAs levels [21] might raise the question whether a stop in taking their antidepressant medications could lead to even lower BCAAs levels in depressive patients than those already observed in this present study. Additional studies including depressive patients free of antidepressants would be valuable. Knowledge about the causal biochemical pathways explaining the reduced BCAAs levels in depressive patients is still very limited and must be explored in the future.

The reported observation that fluoxetine and methylphenidate induce mTor activity [30] supports the theory that mTor might play a crucial role in the pathophysiology of major depression. In this case low mTOr activity, due to decreased levels of BCAAs, might provoke or further aggravate this pathophysiological state. However, the direct measurement of the mTOR complex is rather difficult. It is currently only possible in tissue cells after electrophoretic separation and western blotting. If a simple analysing system becomes available in the future we will aim to carry out follow-up studies. Thus, further studies are required to determine any set patterns. Knowledge about the complex interaction between BCAAs and mTor is still limited and requires ongoing research.

Finally, additional studies might address the question, if the effects observed might be reversible after clinical improvement of depressive symptomatology in patients with major depression.

## Conclusions

A reduced activation of mTor due to a reduction of BCAAs could play a crucial and unrecognised factor in the etiology of depression and may provoke depressive symptomatology and lower energy metabolism in patients with major depression. In the future, mTor and its up- and downstream signalling partners might be important targets for the development of novel antidepressants. However, not only biological pathways but also psychological variables might provoke severe mental illness and reduced health-related quality of life in patients suffering from major depression. Thus the synopsis of all contributing factors into one holistic biopsychosocial model is the main scientific goal in the future [32].

## Supporting Information

S1 Dataset (XLSX)

## Abbreviations

BDI-II: Beck Depression Inventory-II
BCAAs: Branched-Chain Amino Acids
HAMD-17: Hamilton Rating Scale for Depression
mTor: Mammalian Target of Rapamycin
SD: Standard Deviation
SNRI: second-generation dual-action antidepressants
SSRI: selective serotonin re-uptake inhibitors
